# *RPS4Y *gene family evolution in primates

**DOI:** 10.1186/1471-2148-8-142

**Published:** 2008-05-13

**Authors:** Olga Andrés, Thomas Kellermann, Francesc López-Giráldez, Julio Rozas, Xavier Domingo-Roura, Montserrat Bosch

**Affiliations:** 1 Genètica de la Conservació Animal, Institut de Recerca i Tecnologia Agroalimentàries, Crta. de Cabrils km2, 08348 Cabrils, Spain; 2 Departament de Ciències Experimentals i de la Salut, Universitat Pompeu Fabra, Dr. Aiguader 80, 08003 Barcelona, Spain; 3 Institut für Immungenetik, Charité-Universitätsmedizin Berlin, Campus Benjamin Franklin, Freie Universität Berlin, Thielallee 73, 14195 Berlin, Germany; 4 Departament de Genètica, Universitat de Barcelona, Av. Diagonal 645, 08028 Barcelona, Spain

## Abstract

**Backgound:**

The *RPS4* gene codifies for ribosomal protein S4, a very well-conserved protein present in all kingdoms. In primates, *RPS4* is codified by two functional genes located on both sex chromosomes: the *RPS4X *and *RPS4Y *genes. In humans, *RPS4Y *is duplicated and the Y chromosome therefore carries a third functional paralog: *RPS4Y2*, which presents a testis-specific expression pattern.

**Results:**

DNA sequence analysis of the intronic and cDNA regions of *RPS4Y *genes from species covering the entire primate phylogeny showed that the duplication event leading to the second Y-linked copy occurred after the divergence of New World monkeys, about 35 million years ago. Maximum likelihood analyses of the synonymous and non-synonymous substitutions revealed that positive selection was acting on *RPS4Y2 *gene in the human lineage, which represents the first evidence of positive selection on a ribosomal protein gene. Putative positive amino acid replacements affected the three domains of the protein: one of these changes is located in the KOW protein domain and affects the unique invariable position of this motif, and might thus have a dramatic effect on the protein function.

**Conclusion:**

Here, we shed new light on the evolutionary history of *RPS4Y *gene family, especially on that of *RPS4Y2*. The results point that the *RPS4Y1 *gene might be maintained to compensate gene dosage between sexes, while *RPS4Y2 *might have acquired a new function, at least in the lineage leading to humans.

## Background

*RPS4 *genes encode for the ribosomal protein small subunit 4 (29kD; 263 amino acids), a protein involved in mRNA binding and located at the 40S/60S subunit interface of the small ribosomal subunit [1]. The RPS4 protein is well-conserved in prokaryotes and eukaryotes, which suggests strong functional constraints on structural evolution [2].

*RPS4 *is found on autosomes in all vertebrates except mammals, which all have an X-linked copy (*RPS4X*). Fisher et al. [3] found a Y-linked copy (*RPS4Y*) in humans, and Omoe and Endo [4] postulated that *RPS4Y *was primate specific. However, this study was performed using only great apes and rodents. Moreover, Jegalian and Page [5] found a Y-linked copy in a marsupial species, the gray short-tailed opossum (*Monodelphis domestica*), and Skaletsky et al. [6] found this gene in the first X-degenerate block suggesting that *RPS4Y1 *was present before mammalian radiation. Recently, another Y-linked copy has been discovered on the human Y chromosome and has been named *RPS4Y2 *[6] in order to distinguish it from the first copy, which is now called *RPS4Y1*. The existence of two paralogous copies is a unique feature of human RPS4 compared to other ribosomal proteins [3], and the presence of three copies is even more surprising. This characteristic is also present in *Pan troglodytes*, based on Ensembl information [7]. The maintenance of *RPS4Y *copies in the genome is unexpected as it would destroy the equimolarity among ribosomal proteins described by Meyuhas et al. [8]. These authors showed that the expression of ribosomal protein genes must be regulated in a coordinated way in order to ensure the correct assembly of the elements of the ribosomal complex.

*RPS4Y1 *is ubiquitously expressed and it is located in position p11.31 [3]. Watanabe et al. [9] demonstrated that this gene is functional and functionally interchangeable with *RPS4X *and, despite the lower expression level of *RPS4Y*, both copies appeared to be necessary for correct development [10,11]. Thus, RPS4X and RPS4Y proteins are both found in primate male ribosomes while primate female *RPS4X *genes escape inactivation. Bergen et al. [2] found an increased substitution rate in great ape *RPS4Y1 *than in the X-linked copies, showing fewer functional constraints on the Y genes.

*RPS4Y2 *shows a testis-specific expression pattern in human lineage [[6]; Rozen, personal communication] and it is located in position q11.223, a region associated with infertility (AZFb). However, nothing is yet known about *RPS4Y2 *essentiality, or about its functionality, expression pattern in non-human primates, or the mechanisms associated with its survival. It might be possible that *RPS4Y2 *gene had accumulated mutations that would have improved an extra-ribosomal function already present in the gene since it has been described that ribosomal proteins can perform other functions in addition to their role in the protein synthesis [12]. In fact, Fisher et al. [3] suggested that haploinsufficiency in RPS4 could contribute to Turner syndrome. This, in turn, led Wool [12] to postulate that RPS4 could be involved in the regulation of development.

Here we describe the evolutionary history of *RPS4Y *genes in primates. The study was conducted by analyzing DNA sequences from different species covering the entire primate phylogeny. Our aim was to elucidate the evolutionary mechanisms operating in the retention of these genes and the possible role of positive selection in their evolution. We also estimated the age of the duplication event. Finally, we have discussed the functional implications of RPS4Y2 protein evolution.

## Results

**Figure 1:**
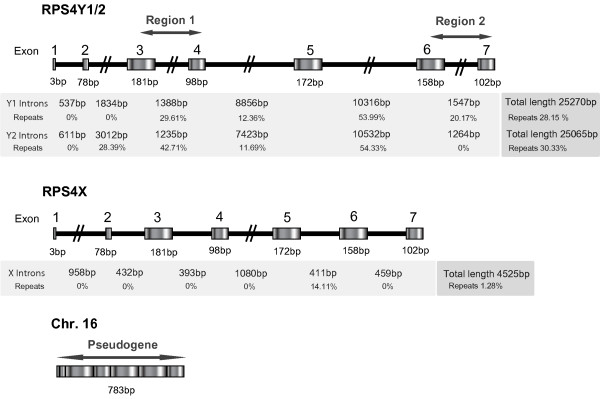
**Genomic structure of human *RPS4 *genes. **Exonic and intronic lengths and repeat content are shown. Amplified loci on the Y chromosome (regions 1 and 2) and on the pseudogene are shown with arrows.

Genomic sequences from *RPS4X, RPS4Y1 *and *RPS4Y2 *genes were obtained from Ensembl. Surprisingly, human and chimpanzee *RPS4 *genes on the Y chromosome were approximately 5 times longer than *RPS4X *(fig. 1), which is extraordinarily extended for a ribosomal gene (25 kb); Yoshihama et al. [13] showed that *RPS4Y *is, in fact, the largest ribosomal protein gene in the human genome. Despite these differences, all three *RPS4 *genes had the same number of exons and these were exactly of the same length. The described protein domains -S4, ribosomal-S4e and KOW- were also conserved. Length variations among *RPS4 *copies were explained by different repeat content in intronic regions. When Repeatmasker was applied, *RPS4X *evidenced hardly any interspersed repeats (< 1.3%), while for *RPS4Y1 *and *RPS4Y2*, about 30% of the sequence corresponded to repeat elements (Fig. 1). Long introns cause a reduction in the level of expression since transcription cost increases, therefore purifying selection serves to maintain intron length [14,15]. A possible reduction in expression levels might explain why only 15% of the RPS4 proteins found in male ribosomes are from the *RPS4Y1 *gene [10]. Besides *RPS4 *genes, a *RPS4Y *retrotransposed pseudogene, containing the 7 exons but disrupted by stop codons, was also detected on both human chromosome 16 and its chimpanzee homologue (Fig. 1).

**Figure 2:**
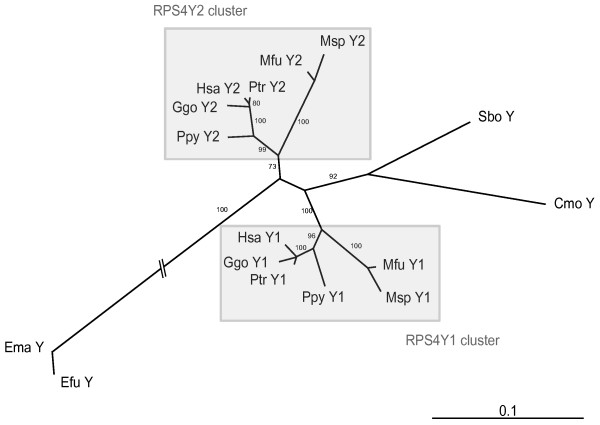
**Neighbor-joining tree with model TVM+G of concatenated region1 and region2 for *RPS4Y1 *and *RPS4Y2 *built with PAUP after GBlocks region selection (787 bp).** Cja was excluded when performing the analyses since its sequence was too short. Boostrap values (10,000 replicates) are shown on each branch.

**Figure 3:**
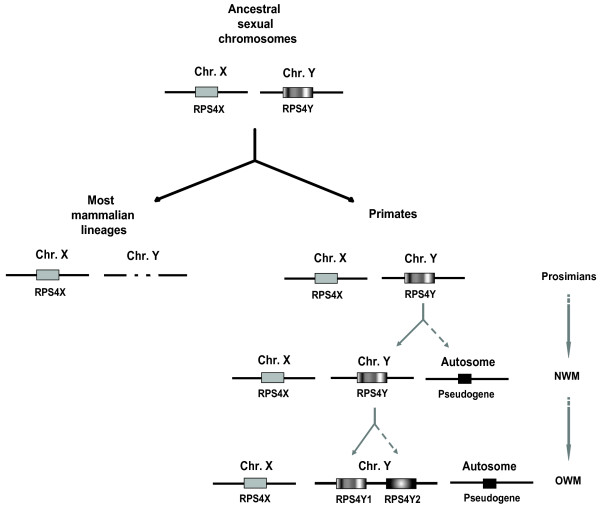
Scheme of mammalian *RPS4 *evolutionary history.

We were able to amplify the *RPS4Y1 *and *RPS4Y2 *introns 3 and 6 in all great apes analyzed (*Pan troglodytes *(Ptr), *Gorilla gorilla *(Ggo), *Pongo pygmaeus *(Ppy)) and Old World monkeys (*Macaca fuscata *(Mfu), *Mandrillus sphinx *(Msp)). New World monkeys (*Saimiri boliviensis *(Sbo), *Callithrix jacchus *(Cja), *Callicebus moloch *(Cmo)) and strepsirrhines (*Eulemur fulvus *(Efu), *Eulemur macaco *(Ema)) produced sequences from only a single copy. This pattern was supported by FISH analyses as two signals could be detected in great apes and OWM Y-chromosome while NWM Y-chromosome presented hybridization signal in one single site (data not shown). The phylogenetic tree from concatenated DNA sequences of introns 3 and 6 showed two different well-defined clusters, one for each duplicated gene copy, suggesting the independent evolution of *RPS4Y1 *and *RPS4Y2 *after the duplication event. NWM and strepsirrhine RPS4Y copies stayed out of the clusters (Fig. 2). When we analyzed intron 3 and intron 6 independently, we obtained similar results. In addition, from the synonymous substitutions of the intronic sequences we estimated that the duplication event probably took place about 35 million years ago, which fitted in with the time between NWM and OWM divergence, estimated at between 25 and 40 million years ago [16]. All these results therefore indicate that *RPS4Y *duplication should have been generated after the divergence of NWM (Fig. 3).

Pseudogene sequences were identified in great apes, OWM and NWM, but not in strepsirrhines. These findings suggested that the *RPS4Y *pseudogene was generated after the divergence of strepsirrhines but before the divergence of NWM. Since NWM presented these sequences, it can be inferred that the pseudogene derived from the ancestral *RPS4Y *copy.

**Figure 4:**
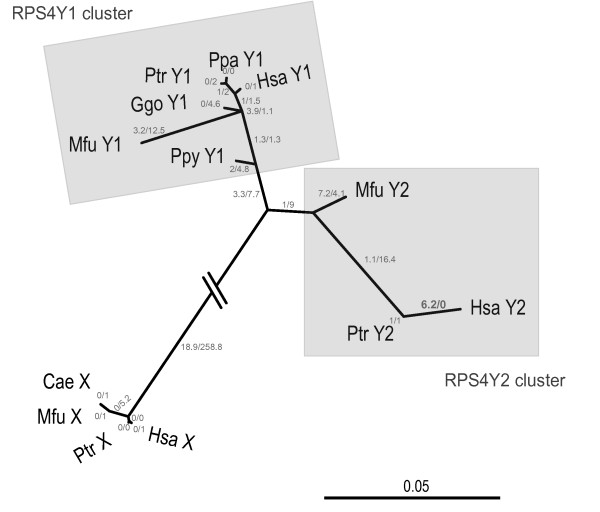
**Neighbor-joining tree with model TrNef+G of *RPS4X*, *RPS4Y1*, *RPS4Y2 *cDNAs built with PAUP.** Numbers of non-synonymous and synonymous substitutions are shown on each branch. The sequences analyzed were 789 bp long.

*RPS4Y1*, *RPS4Y2 *and *RPS4X *GenBank cDNA sequences from great apes and OWM produced a phylogenetic tree with 3 distinct clusters (Fig. 4). When we included pseudogene sequences from this study in the analysis, a phylogenetic tree with 4 well-defined clusters was generated; *RPS4X *sequences constituted a distantly separated cluster while pseudogene sequences were included in *RPS4Y1 *group (data not shown).

We conducted a number of relative rate tests to detect putative deviations from the molecular clock expectations. For cDNA sequences, the results suggested that, using *RPS4X *sequences as the outgroup, only combinations involving pseudogene sequences produced statistically significant results (p-value < 0.04). With respect to intronic concatenated sequences, macaque was the only lineage to generate differences when comparing *RPS4Y2 *sequences and using NWM sequence as the outgroup (p-value < 0.04). The same results were obtained after the FDR correction for multiple tests (data not shown).

To determine the effect of natural selection on coding gene regions, we applied a number of maximum likelihood codon models implemented in the PAML software package [17]. To control the false discovery rate for multiple tests we applied the method described in [18].

### Branch models

We applied the one ratio (M0) model -which assumes the same ω ratio for the complete tree- and the free-ratio (FR) model -which allows for different ω ratios across tree-branches. We compared the two models using likelihood ratio test (LRT) to determine whether our data was compatible with homogeneous selective pressure across the branches. Whenever RPS4Y2 sequences were considered, FR fitted the data significantly better than M0. Yet for RPS4Y1 and RPS4X, the M0 model could not be rejected (Table 1). When human sequences were not considered in the analyses, M0 fitted the data better than FR, even when RPS4Y2 sequences were included (Table 1), showing that the human RPS4Y2 lineage has evolved under a different ω ratio. To confirm this result, we also applied the two-ratio model (M2b) -which allows for different ω ratios in selected branches of the tree- and compared this with M0 model. We chose human and macaque *RPS4Y2 *as possible foreground branches based on the exclusive amino acid substitutions of each protein observed in the protein alignment (see Additional file 1) and the distribution of non-synonymous and synonymous substitutions shown in Fig. 4. M2b fitted the data better than M0 in both analyses (data not shown). However, only ω value for human *RPS4Y2 *was greater than 1 (ω_Hsa _could not be estimated since there were no synonymous substitutions), showing the action of positive selection in this branch. Macaque *RPS4Y2 *has evolved at a greater ω than the other branches (0.6147 and 0.0581, respectively), except for human *RPS4Y2*, which could indicate a relaxation of purifying selection in this branch (data not shown).

**Table 1:** Parameter estimates for the one ratio and free-ratio branch models.

Data Set	Model	*f*	l	Estimated parameters	*κ*
*RPS4Y1*	One ratio (M0)	11	-1275.00	ω = 0.161	3.40
	Free-ratio (FR)	19	-1269.87	Ggo ω = 1.27	3.39
*RPS4Y2*	One ratio (M0)	5	-1239.64	ω = 0.278	3.78
	Free-ratio (FR)*	7	-1233.58	Hsa^a^	3.77
*RPS4X*	One ratio (M0)	7	-1100.83	ω = 0.0001	--- b
	Free-ratio (FR)	11	-1100.83	Ptr ω = 15.81	--- b
*RPS4Y1/Y2*	One ratio (M0)	17	-1571.22	ω = 0.192	4.55
	Free-ratio (FR)*	31	-1555.16	Ptr Y1 ω = 1.71; Hsa Y2^a^; Ggo/Mfu Y1 ω = 1.41	4.55
*RPS4Y1/Y2/X*	One ratio (M0)	25	-1994.26	ω = 0.069	2.95
	Free-ratio (FR)*	47	-1961.77	Hsa Y2^a^; Ggo/Mfu ω = 1.30	3.29
*Y*1*Y*2*X*	One ratio (M0)	23	-1937.8	ω = 0.058	2.95
No Hsa *Y*2					
	Free-ratio (FR)	43	-1917.62	Ggo/Mfu ω = 1.30	3.24
*Y*1*Y*2	One ratio (M0)	15	-1518.97	ω = 0.1520	4.55
No Hsa *Y*2					
	Free-ratio (FR)	27	-1510.59	Ppa ω = 1.25; Ggo/Mfu Y1 ω = 1.46	4.56
*Y*1*Y*2*X *Hsa	One ratio (M0)	5	-1630.42	ω = 0.051	2.85
	Free-ratio (FR)*	7	-1620.58		3.14

*f *for the number of free parameters; l for likelihood values; and *κ *for transitions/transversions

* Posterior probability >0.95.

^a ^ω cannot be estimated since there are no synonymous substitutions.

^b ^κ cannot be estimated since there are no transversions.

### Site models

Site-specific likelihood models [19,20] make it possible to detect variable selective pressures across sites. Three nested models were performed: M1 (neutral) and M2 (selection); M0 (one ratio) and M3 (discrete); and M7 (beta) and M8 (beta&ω). None of the LRT tests was significant; we therefore found no evidence for sites evolving under positive selection across the different species.

### Branch-site models

If positive selection only affects certain sites of specific lineages, previous models would probably not have been able to detect them. We therefore applied the more realistic branch-site models A and B [21], which allow ω ratio to vary among sites and lineages. Test 1 for Model A compared this model with the site-specific model M1 (neutral); test 2 compared model A against null model A. Model B was compared with the site-specific model M3 (discrete, *K *= 2). As above, we chose human and macaque *RPS4Y2 *as foreground branches. We found that models A (test 1) and B only fitted the data significantly better than simpler models when human *RPS4Y2 *lineage was used as the foreground branch (Table 2 for model A); this indicates that positive selection acted on some sites of the human *RPS4Y2 *gene. Test 2 confirmed this result when *RPS4Y1 *and *RPS4Y2 *sequences were considered.

**Table 2:** Parameter estimates for branch-site model A from maximum likelihood analyses.

Data Set	Foreground branch	*f*	l	Estimated parameters	*κ*	Positive selection (BEB)
*RPS4Y1*	Hsa *Y*1	14	-1275.00	p_0 _= 1 p_1 _= 0 (p_2_+p_3 _= 0), ω_2 _= 1	3.40	
*RPS4Y2*	Hsa *Y*2	8	-1233.73	p_0 _= 0 p_1 _= 0 (p_2_+p_3 _= 1), ω_2 _= 139.62	3.78	68H*, 70L*, 87I*, 108C*, 185A*
*RPS4X*	Hsa *X*	10	-1100.83	p_0 _= 1 p_1 _= 0 (p_2_+p_3 _= 0), ω_2 _= 1	--- ^b^	
*RPS4 Y*1/*Y*2	Hsa *Y*2	20	-1562.15	p_0 _= 0 p_1 _= 0 (p_2_+p_3 _= 1), ω_2 _^a^	4.61	70I*, 185G*
	*Y*2cluster	20	-1570.33	p_0 _= 0 p_1 _= 1 (p_2_+p_3 _= 1), ω_2 _= 139.62	4.59	
*RPS4 Y*1/*Y*2/*X*	Hsa *Y*2	28	-1971.13	p_0 _= 0 p_1 _= 0 (p_2_+p_3 _= 1), ω_2 _^a^	3.21	68R*, 70I*, 87M*, 104D*, 108R*, 185G*
	*Y*2cluster	28	-1986.19	p_0 _= 0.942 p_1 _= 0.058 (p_2_+p_3 _= 0), ω_2 _= 1	3.12	
*Y*1*Y*2*X *No Hsa *Y*2	*Y*2cluster	26	-1925.91	p_0 _= 0.939 p_1 _= 0.061 (p_2_+p_3 _= 0), ω_2 _= 1	3.16	
*Y*1*Y*2 No Hsa *Y*2	*Y*2cluster	18	-1516.95	p_0 _= 0.901 p_1 _= 0.099 (p_2_+p_3 _= 0), ω_2 _= 1	4.62	
*Y*1*Y*2*X *Hsa	Hsa *Y*2	8	-1619.84	p_0 _= 0.647 p_1 _= 0.021 (p_2_+p_3 _= 0.332), ω_2 _= 1	3.03	68R, 70I, 87M, 104D, 108R, 180L, 185G, 205F, 222L (P > 0.71)

*f *for the number of free parameters; l for likelihood values; and *κ *for transitions/transversions.

* Posterior probability >0.95.

^a ^ω cannot be estimated since there are no synonymous substitutions.

^b ^κ cannot be estimated since there are no transversions.

When only *RPS4Y2 *sequences were considered, Bayes Empirical Bayes (BEB) analysis in model A identified the amino acids located at positions 68, 70, 87, 108 and 185 as positively selected sites. When *RPS4Y1 *sequences were also analyzed, only amino acids at positions 70 and 185 were chosen as positively selected. Finally, when all the sequences were considered (*RPS4Y1*, *RPS4Y2 *and *RPS4X*) the same 5 positions plus amino acid 104 were identified as putative targets of positive selection (Table 2). All positions were still significant after the correction for multiple tests (data not shown). Positions 70 and 185 were identified in all sequence sets and had the highest posterior probabilities; moreover, these two positions were corroborated by conservative test 2. Amino acid replacements in the human *RPS4Y2 *lineage involved all 3 RPS4 protein domains (S4, Ribosomal-S4e and KOW) (Table 3; see Additional file 1).

**Table 3:** Positive positions selected by Bayes Empirical Bayes analysis in model A of the PAML.

			Change type				
		
BEB	Ancestral amino acid	Hsa RPS4Y2	Charge	Polarity	Polarity & Volume	Data Sets	Domain affected
68*	R	H	Cons.	Cons.	Cons.	a, c	S4
70*	I	L	Cons.	Cons.	Cons.	a, b, c	S4
87*	M	I	Cons.	Cons.	Cons.	a, c	S4
104*	D	N	Rad.	Cons.	Cons.	c	Ribosomal-S4E
108*	R	C	Rad.	Cons.	Rad.	a, c	Ribosomal-S4E
185*	G	A	Cons.	Rad.	Cons.	a, b, c	KOW

Data Sets: a) for RPS4Y2; b) for RPS4Y1 and RPS4Y2; and c) for RPS4Y1, RPS4Y2 and RPS4X.

Cons. for conservative change; Rad. for radical change.

* Posterior probability >0.95.

We compared the number of synonymous and non-synonymous substitutions in each human *RPS4 *gene family branch (FR model, using *RPS4X *as the ancestral sequence) to determine the putative correlation expected under the neutral model. We did not found significant differences between *RPS4Y1 *and *RPS4Y2 *(Fisher exact test, p = 0.54), although the two Y-linked *RPS4 *copies evolved at different rates than the X copy (p < 0.0005).

To study in more detail the evolution of the human lineage we estimated the nucleotide divergences between human and chimpanzee *RPS4Y1*, *RPS4Y2 *and *RPS4X *cDNA, intronic and pseudogene sequences (Table 4). It should be highlighted that the *K*_a_/*K*_s _ratio of *RPS4X *was zero due to the absence of non-synonymous changes; this was a result of the strong functional constraints in this gene. As far as *RPS4Y1 *was concerned, the increase in both the *K*_a_/*K*_s _ratio (ω = 0.062) and *K*_s _(0.0270) with respect to *RPS4X *pointed to a relaxation in purifying selection. This relaxation could also be observed in the intronic sequences, as the intronic divergence estimate (*K*_i_) was higher for *RPS4Y1 *(and similar to *RPS4Y2 *and pseudogene, with *K*_i _values of 0.0142, 0.0152 and 0.0181, respectively) than for *RPS4X *(*K*_i _= 0.0076). Finally, and as expected, *RPS4Y2 *exhibited a *K*_a_/*K*_s _ratio larger than 1 (ω = 2.9477) due to the elevated *K*_a _value (0.0129) since the *K*_s _value (*K*_s _= 0.0044) was similar to that of *RPS4X *(*K*_s _= 0.0038). These results could not be explained by a relaxation in purifying selection (i.e. by a reduction in functional constraints) and pointed towards the action of positive selection in human *RPS4Y2 *reinforcing the former PAML analyses.

**Table 4:** Nucleotide divergence of Hsa and Ptr sequences estimated by pairwise comparisons using DnaSP and PAML software.

	DnaSP			PAML		
		
Data Set	*K*_a_	*K*_s _(*K*_i_)	*K*_a_/*K*_s_	*K*_a_	*K*_s_	*K*_a_/*K*_s_
cDNA *Y*1	0.0017	0.0270	0.0619	0.0018	0.0224	0.0815
cDNA *Y*2	0.0118	0.0053	2.2147	0.0129	0.0044	2.9477
cDNA *X*	0	0.0053	0	0	0.0038	0
Introns *Y*1	-	0.0142	-			-
Introns *Y*2	-	0.0152	-			-
Introns *X*	-	0.0076	-			-
Pseudogene	-	0.0181	-			-

## Discussion

First efforts to describe mammalian *RPS4 *phylogeny suggested that *RPS4 *moved to the X chromosome before mammalian radiation while *RPS4 *Y-linked copy was primate specific [2]. However, the discovery of a *Rps4 *Y-linked copy in the non-primate species *M. domestica *[5] and the location of human *RPS4Y *in an X-degenerate block [6] suggest that *RPS4X *and *RPS4Y *were present in the ancestral mammalian sex chromosomes but were lost in the Y-chromosome of most lineages during the mammalian evolution [22]. Here we have demonstrated that species along all primate phylogeny maintained the *RPS4Y1 *gene and that the second Y-linked copy was originated from the duplication of the *RPS4Y1 *gene after the divergence of NWM but before the radiation of OWM (Fig. 3 shows a scheme of *RPS4 *evolution in mammals).

The loss of *RPS4Y1 *gene in most mammals is a result of the degeneration, in both size and gene content, of the Y-chromosome during evolution. It seems that most of the genes retained are related to male-specific functions [23]. Then, the maintenance of ribosomal proteins in primate Y chromosome is unexpected and the mechanisms operating under their retention are still unknown. Since it has been demonstrated that *RPS4Y1 *maintains the ribosomal activity and it is essential for viability, a possible explanation for its preservation might be the compensation of gene dosage between sexes. Since genes on the X chromosome are inactivated to overcome sex differences, ribosomal proteins on the X chromosome need a mechanism to achieve equimolarity with other ribosomal proteins. In non-primate mammals, the active *RPS4X *in females and the sole *RPS4X *in males therefore need to be more fully expressed than autosomal genes. The existence of a functional Y-linked copy in primates has led *RPS4X *to escape inactivation, as *RPS4Y *entails gene dosage compensation. In fact, in humans, the other three ribosomal X chromosome protein genes (*RPL10*, *RPL36A*, and *RPL39*) achieve equimolarity by using functional processed copies (*RPL10L*, *RPL36AL*, and *RPL39L*) elsewhere in the genome [24]. However, this hypothesis could not explain the maintenance of the *RPS4Y2 *gene.

Gene duplication is a major force for the rise of new gene functions in evolution. The average rate of gene duplication is 0.01 per gene per million years [25]. However, the most common fate of duplicate pairs is that one of the copies becomes a pseudogene by the fixation of deleterious mutations, and will finally be lost in the genome. Half-lives of duplicated genes that finally disappear tend to range from 1 to 17 million years [25]. We have shown that *RPS4Y2 *emerged in the primate phylogeny between the divergence of NWM and OWM. Despite the fact that the expression pattern of *RPS4Y2 *in non-human primates is still unknown, the absence of stop codons in the open reading frame suggests that the gene is still active, at least in Mfu and Ptr. We can therefore undoubtedly discard a pseudogenization process since the gene has remained in the genome for approximately 35 million years.

On the other hand, duplicated genes can be retained in the genome after neofunctionalization – when a completely new function is acquired [26] – or subfunctionalization – when either the two genes become specialized in different tissues or at different developmental stages [27] or when the ancestral gene had several functions and the duplicates become specialized for certain of these functions. It has been found that positive selection plays an important role in duplicated gene retention in mammalian genomes [28] and is active in both events. The testis and prostate-specific expression found in human *RPS4Y2 *points to a subfunctionalization event. This hypothesis is supported by the fact that the human *RPS4Y2 *promoter presents the oligopyrimidine tract as being disrupted by a mutation (data not shown). This tract is the only feature present in all ubiquitously expressed human ribosomal proteins [13] and its disruption would account for the specificity of *RPS4Y2 *expression in humans. Interestingly, the oligopyrimidine tract of the promoter in chimpanzee *RPS4Y2 *has remained untouched, which would suggest ubiquitous expression in this species. Studies of *RPS4Y2 *expression patterns in all primate lineages are needed to elucidate whether this expression is testis-specific as in humans or ubiquitous as suggested by our observations relating to the chimpanzee *RPS4Y2 *promoter. If human-specific testis restricted expression is confirmed, *RPS4Y2 *would compile all the characteristics mainly associated with human speciation – testis-specific expression and human-specific expression pattern and function [29].

However, the detection of positive selection and the relaxation of purifying selection suggest that *RPS4Y2 *copy has either undergone a neofunctionalization process or been subjected to a functional specialization, at least in the human lineage. We found that model A (test 1) pointed to six positively-selected positions, while test 2, which is more powerful, but very conservative, confirmed positive selection in only two of the amino acid positions. Since current statistical methods are very conservative at the moment of detecting weak positive selection, some of the other positions identified by test 1 may also have been affected by positive selection.

It is not clear which specific activity of RPS4Y2 could be affected since the six positively-selected amino acids involve all of the protein domains. There were three amino acids in the S4 domain (amino acids 68, 70, 87), two in the ribosomal_S4E domain (104, 108), and one in the KOW domain (185). All changes in the S4 domain were conservative, while changes in the ribosomal_S4E and KOW domains were radical. Moreover, the amino acid affected by positive selection in the KOW domain of RPS4Y2 is the only residue conserved in the KOW motif – a glycine in position 11 – [30]. In human RPS4Y2, this invariable glycine residue has been replaced by an arginine, and so the function of the domain may be dramatically affected. Mutations in human *RPS4Y2 *gene, therefore, may have improved an extra-ribosomal function that was already present in the ancestral gene. On the other hand, *RPS4Y2 *location in the azoospermia region AZFb – where large microdeletions have been described to cause azoospermia, even if the genes responsible for this phenotype have not yet been identified [31] – and its testis-specific expression pattern point to a possible connection between RPS4Y2 and fertility. These features suggest that RPS4Y2 may have acquired a new spermatogenesis-related function in human male lineage; this would be consistent with the observed excess of sperm-specific genes affected by positive selection [32]. In order to elucidate the putative effects of the amino acid changes on the protein function, its interactions within the ribosomal complex, and the binding to RNA, it is necessary to carry out further functional and biochemical studies. It is especially interesting to elucidate if RPS4Y2 maintains the ribosomal protein function. If so, this is the first time that positive selection acting on ribosomal protein is unambiguously demonstrated. Only one recent study [33] suggested that three ribosomal protein genes (one in the human genome and two in the chimpanzee genome) might be positively selected, but signs of positive selection were weak and it was not possible to distinguish between positive selection and a relaxation of selective constraints. Complementation analyses of a rodent *Rps4 *knockout mutant with human *RPS4Y2 *gene would help to elucidate if this gene still conserves its original function or whether, according to the evidences presented in this work, it has acquired a functional specialization related to an extra-ribosomal function or even a new function. Moreover, studies to correlate testis histopathology with different combinations of loss of genes located in AZF regions would reveal if RPS4Y2 has acquired a fertility-related function.

## Conclusion

In conclusion, using comparative sequence analyses, we were able to establish the genealogy of *RPS4Y *genes in primate phylogeny, corroborating the preservation of the first *RPS4Y *gene in all primate infraorders and dating the origin of *RPS4Y2 *as occurring between the divergence of NWM and OWM. *RPS4Y1 *maintenance seems to be the result of a mechanism for compensating gene dosage between sexes. On the other hand, we detected that the human *RPS4Y2 *gene evolved under positive selection. The results and evidences presented here point to the acquisition of a non-ribosomal function -an extra-ribosomal or a completely new male-specific function- of the *RPS4Y2 *in the human lineage.

## Methods

### Samples

Samples of *Homo sapiens *(Hsp), great apes -*Pan troglodytes *(Ptr), *Gorilla gorilla *(Ggo), and *Pongo pygmaeus *(Ppy) -, Old World monkeys (OWM) -*Macaca fuscata *(Mfu), and *Mandrillus sphinx *(Msp) -, New World monkeys (NWM) -*Saimiri boliviensis *(Sbo), *Callithrix jacchus *(Cja), and *Callicebus moloch *(Cmo) -, and strepsirrhines -*Eulemur fulvus *(Efu), and *Eulemur macaco *(Ema)- were provided from the INPRIMAT sample collection. For tissue and blood samples DNA was extracted using the Qiagen tissue kit (Qiagen, Valencia, CA, USA) following the manufacturer's instructions. Initial amounts were 25 mg for muscle tissues and 100 μl for blood samples. DNA from cell lines was also provided by INPRIMAT DNA collection (see Additional file 2).

### Amplification and sequencing

Intron 3 and intron 6 of *RPS4Y *genes were found suitable for amplification, since they allowed the location of both forward and reverse primers on exonic sequences to amplify the full intron (Fig. 1). Primers were designed to be male specific and to distinguish between *RPS4Y2 *and *RPS4Y1 *in different primate species. We also designed another pair of primers to amplify a complete 7-exon mRNA *RPS4Y *pseudogene (Fig. 1). The names and sequences of the oligonucleotides are shown in Additional file 3. We have described the PCR conditions, fragments resulting from the use of different primer combinations, and species specificity in Additional file 4.

PCR products were purified using the GFX PCR DNA and Gel Band Purification Kit (Amersham Biosciences UK Limited, Buckinghamshire, UK). Both strands were sequenced from the purified products using forward and reverse PCR primers (sequencing conditions are described in Additional file 4).

### Sequence analysis

Genomic sequences and information concerning human and chimpanzee *RPS4 *genes were taken from Ensembl [7]. We used the RepeatMasker v3.1.6. program [34] to detect interspersed repeats in Ensembl genomic sequences.

We handled DNA sequences from this study and protein and cDNA sequences obtained from GenBank (see Additional files 5 and 6 for accession numbers) with BioEdit v6.0.7 [35]. Multiple alignments were obtained by Clustal W [36] or DiAlign2 [37] and subsequently manually edited to minimize the number of gaps.

Once aligned, we applied Gblocks [38] to eliminate poorly aligned positions and divergent regions of our intronic sequences in order to obtain reliable blocks that were suitable for phylogenetic analysis. We used DnaSP v4.0 [39] to estimate nucleotide diversity and descriptive statistics to examine the different sequence sets.

For each alignment, we selected the nucleotide substitution model that best fitted the data among 56 different evolutionary models based on the Akaike Information Criteria approach using Modeltest 3.6 [40]. We constructed a phylogenetic tree (Fig. 2) based on the neighbor-joining (NJ) method [41], using PAUP*v4.0b10 [42]. Confidence in the resulting relationships was assessed using 10,000 bootstrap replicates [43]. We also performed relative rate tests on the trees by applying the RRTree program [44], which compares substitution rates between lineages of DNA sequences, relative to a particular outgroup. TreeView [45] was used to visualize trees.

Analysis of the impact of positive or negative selection on DNA coding region was conducted using Phylogenetic Analysis by Maximum Likelihood (PAML) v3.12 [17] and DnaSP [39] software. We applied the different codon substitution models implemented in codeml (Branch Models, Site Models and Branch-Site Models). We applied a FDR correction for multiple tests in all analyses.

We estimated the time of the duplication event (Td) from the mean number of synonymous substitutions per site (${\bar{\text{K}}}_{\text{s}}$) among all paralogous combinations. For each paralogous copy, the synonymous substitution rate (*r*) was estimated for all possible pairs of species, as *r *= *K*_s_/2*Ts, where *K*_s _is the number of synonymous substitutions per site and Ts is the divergence time for each pair of species. For Ts, we took the minimum and maximum values from Goodman et al. [16]. Average rates (*ř*) for both the minimum and maximum values were obtained from the slope of a regression analysis. We then applied the equation Td = ${\bar{\text{K}}}_{\text{s}}$/2*ř to find the estimated duplication time range.

## Authors' contributions

OA conceived and participated in the design of the study, participated in sequence generation, performed sequence and statistical analyses and drafted the manuscript. TK conceived and participated in the design of the study, participated in sequence generation and FISH analyses and helped in an early draft of the manuscript. FL-G helped in sequence and statistical analyses. JR helped in sequence and statistical analyses and in the draft of the manuscript. XD-R conceived and participated in the design of the study. MB participated in the design of the study, carried out FISH analyses and coordinated and helped in the draft of the manuscript. All authors read and approved the manuscript.

## Supplementary Material

Additional file 1 **Additional figure 1:** Primate RPS4 protein alignment. Four different Pfam domains are marked in different colors. Nucleotide positions under positive selection in the human RPS4Y2 lineage are highlighted in red squares. Click here for file

Additional file 2 **Supplementary table 1.** Samples information: INPRIMAT code, species name, sex and DNA source are given. Click here for file

Additional file 3 **Supplementary table 2.** Primers designed to specifically amplify RPS4Y2 and RPS4Y1 in different primate species. Nomenclature: e.g. C1E3F1 = C1 (specific for RPS4Y copy 1), E3 (located in exon3), F (forward), and 1 (first primer designed in this location). CY means primer that amplifies both Y-linked copies. mRNAYF and mRNAYR were used to amplify the pseudogene. Click here for file

Additional file 4 **Supplementary table 3.** Resulting fragments using different primer combinations. E.g. Reg1C1a = Reg1 for region 1 (intron3), C1 for copy 1, a for first fragment amplified. Nomenclature analogue for region 2 (intron6). CY means there is no Y-linked copy specificity. MgCl2 [mM] column shows the concentrations used in the experiments and T_Exp_ is the experimental annealing temperature (TD) means that PCR program included touch down cycles. Three letter code indicate the species amplified with each primer pair. Click here for file

Additional file 5 **Supplementary table 4.** Accession numbers of intronic and pseudogenic sequences generated in this study. Code: Y1 for RPS4Y1, Y2 for RPS4Y2, Y for unique RPS4Y gene, and ψ for pseudogene. Click here for file

Additional file 6 **Supplementary table 5.** Accession numbers of nucleotide and protein sequences used from GenBank for cDNA and protein analyses. Y1 is used for RPS4Y1, Y2 is used for RPS4Y2, and X is used for RPS4X genes. Click here for file
