# Supplementary material for: RPS4Y gene family evolution in primates
Source: BMC Evol Biol. 2008 May 13;8:142. doi: 10.1186/1471-2148-8-142 (PMC2397393; doi:10.1186/1471-2148-8-142)
Supplement: Additional file 2 — Supplementary table 1. Samples information: INPRIMAT code, species name, sex and DNA source are given. [file 1471-2148-8-142-S2.pdf]

Supplementary table 1: Samples information: INPRIMAT code, species name, sex and DNA source are given.

Code	INPRIMAT code	Species	Sex	DNA source
Hsa_M		*Homo sapiens*	Male	Blood
Ptr_M	PTR00321IN	*Pan troglodytes* (Ptr)	Male	Cell line
Ptr_F		*Pan troglodytes* (Ptr)	Female	Cell line
Ggo_M	GGO00605IN	*Gorilla gorilla* (Ggo)	Male	Muscle
Ggo_F		*Gorilla gorilla* (Ggo)	Female	Cell line
Ppy_M		*Pongo pygmaeus* (Ppy)	Male	Muscle
Ppy_F	PPY00329IN	*Pongo pygmaeus* (Ppy)	Female	Cell line
Mfu_M		*Macaca fuscata* (Mfu)	Male	Blood
Mfu_F		*Macaca fuscata* (Mfu)	Female	Blood
Msp_M		*Mandrillus sphinx* (Msp)	Male	Blood
Msp_F		*Mandrillus sphinx* (Msp)	Female	Blood
Sbo_M	SBO00731IN	*Saimiri boliviensis* (Sbo)	Male	Blood
Sbo_F	SBO00688IN	*Saimiri boliviensis* (Sbo)	Female	Blood
Cja_M	CJA00376IN	*Callithrix jacchus* (Cja)	Male	Cell line
Cmo_M		*Callicebus moloch* (Cmo)	Male	Cell line
Efu_M	EFU00661IN	*Eulemur fulvus* (Efu)	Male	Muscle
Ema_M		*Eulemur macaco* (Ema)	Male	Cell line
