# Supplementary material for: RPS4Y gene family evolution in primates
Source: BMC Evol Biol. 2008 May 13;8:142. doi: 10.1186/1471-2148-8-142 (PMC2397393; doi:10.1186/1471-2148-8-142)
Supplement: Additional file 3 — Supplementary table 2. Primers designed to specifically amplify RPS4Y2 and RPS4Y1 in different primate species. Nomenclature: e.g. C1E3F1 = C1 (specific for RPS4Y copy 1), E3 (located in exon3), F (forward), and 1 (first primer designed in this location). CY means primer that amplifies both Y-linked copies. mRNAYF and mRNAYR were used to amplify the pseudogene. [file 1471-2148-8-142-S3.pdf]

Supplementary table 2: Primers designed to specifically amplify *RPS4Y2* and *RPS4Y1* in different primate species. Nomenclature: e.g. C1E3F1 = C1 (specific for *RPS4Y* copy 1), E3 (located in exon3), F (forward), and 1 (first primer designed in this location). CY means primer that amplifies both Y-linked copies. mRNAYF and mRNAYR were used to amplify the pseudogene.

Primer name	Primer sequence 5' to 3'
C1E3F1	CTCAGGAATAGACTCAAGTATGCGT
C2E3F1	CCTCAGGAATAGACTCAAGTATGCA
CYE3F1	TCAGGAATAGACTCAAGTATGCGT
C1E4R1	TCATAGACCAGGCGGAAATGT
C1E4R2	CTTCCACTGTGATGCGGTGA
C2E4R1	GCGGAAATGCTCACCTGTT
C2E4R2	TCTTCCGCTGTGATACGATGA
CYE4R1	CAAAACGGCCCTTGGTGTC
C1E6F1	GGGAAAGACATCCTGGTTCTTTT
C1E6F2	TGTATGGTGATTGGTGGAGCC
C2E6F1	GGAAAGACATCCTGGTTCTTGC
C2E6F2	TGGAGCTAACCTCGGTCGTG
CYE6F1	CTTTTGATGTGGTGCATGTGAAG
C1E7R1	AGCCACTGCTCTGTTTGGTG
C1E7R2	TTAGCCACTGCTCTGTTTGGTG
C2E7R1	GCCACTGCTCTGTTTGGCA
C2E7R2	TCTCTCTTCAGCAATAGTAAGTCGG
CYE7R1	GGCAGGGAAATCCAAGGTTTA
mRNAYF	GGGCCCTAAGAAGCACTTG
mRNAYR	TTAGCCACTGCTGTTTGGTG
