# Supplementary material for: RPS4Y gene family evolution in primates
Source: BMC Evol Biol. 2008 May 13;8:142. doi: 10.1186/1471-2148-8-142 (PMC2397393; doi:10.1186/1471-2148-8-142)
Supplement: Additional file 4 — Supplementary table 3. Resulting fragments using different primer combinations. E.g. Reg1C1a = Reg1 for region 1 (intron3), C1 for copy 1, a for first fragment amplified. Nomenclature analogue for region 2 (intron6). CY means there is no Y-linked copy specificity. MgCl2 [mM] column shows the concentrations used in the experiments and TExp is the experimental annealing temperature (TD) means that PCR program included touch down cycles. Three letter code indicate the species amplified with each primer pair. [file 1471-2148-8-142-S4.pdf]

Supplementary table 3: Resulting fragments using different primer combinations. E.g. *Reg1C1a* = *Reg1* for region 1 (intron3), *C1* for copy 1, *a* for first fragment amplified. Nomenclature analogue for region 2 (intron6). *CY* means there is no Y-linked copy specificity. $MgCl_2$ [mM] column shows the concentrations used in the experiments and T_{Exp} is the experimental annealing temperature (TD) means that PCR program included touch down cycles. Three letter code indicate the species amplified with each primer pair.

Fragment	F Primer	R Primer	$MgCl_2$ [mM]	T_{Exp} (ºC)	Species amplified
Reg1C1a	C1E3F1	C1E4R1	1,5	60 (TD)	Ptr/Ppy/Sbo/Efu
Reg1C1b	CYE3F1	C1E4R2	2	59	Mfu/Msp
Reg1C2a	C2E3F1	C2E4R1	1,5	60 (TD)	Ptr/Ggo/Ppy
Reg1C2b	CYE3F1	C2E4R2	2	59	Mfu/Msp
Reg1CY	CYE3F1	CYE4R1	2	59	Ggo(c1) /Cja/Cmo/Ema
Reg2C1a	C1E6F1	C1E7R1	1,5	60 (TD)	Ptr/Ppy/Mfu
Reg2C1b	C1E6F2	C1E7R2	2	62	Ggo/Mfu
Reg2C2a	C2E6F1	C2E7R1	1,5	60 (TD)	Ptr/Ppy/Mfu
Reg2C2b	C2E6F2	C2E7R2	2	60,5	Ggo/Ppy
Reg2CY	CYE6F1	CYE7R1	1,5	60 (TD)	Sbo/Cja/Efu
mRNAY	mRNAYF	mRNAYR	1,5	57	Hsa/Ptr/Ggo/Ppy/Mfu/Sbo/Cja

We conducted standard PCR procedures in a final volume of 25µl containing a mix with 0.17 uM of primers, 0.32 mM of dNTPs, 2 mM $MgCl_2$, 0.034 U/µl Taq (Ecogene, Barcelona, Spain) and 0.6 ng/µl of DNA. PCR conditions included an initial cycle at 94°C for 5 minutes (min), 30 cycles divided in three steps of 45 seconds (sec) at 94°C, 45 sec at 57-60.5°C and 1 min at 72°C, and a final extension at 72°C for 5 min.

We used a touchdown PCR approach when normal conditions resulted in an unspecific smear or when the product of interest could only be amplified very weakly. In this approach, DNA was amplified using the same conditions as for the standard PCR protocol, but with 1.5mM of $MgCl_2$. Amplification conditions consisted of an initial cycle at 95°C for 5 min followed by 20 cycles divided into three steps of 30 sec at 95°C, 30 sec from 70°C to 60ºC –primer specific annealing temperature was reduced by 0.5°C every cycle– and 30 sec at 72°C; and 20 more cycles divided into three steps of 30 sec at 95°C, 30 sec at 60°C and 30 sec at 72°C to increase product yield, followed by a final extension at 72°C for 5 min.

Standard sequencing reactions contained 10 ng of the purified DNA, 1.6 ng of primer and 2 µl of the ABI Prism™ Big Dye Terminator Cycle Sequencing 3.1 kit in a final volume of 10 µl. Cycling conditions included an initial cycle at 94°C for 4 min, 25

cycles of 10 sec at 96°C, 5 sec at 50°C and 4 min at 60°C. Sequencing was performed on an ABI 3100 automated DNA sequencer (Applied Biosystems, Foster City, CA, USA).
