# Supplementary material for: RPS4Y gene family evolution in primates
Source: BMC Evol Biol. 2008 May 13;8:142. doi: 10.1186/1471-2148-8-142 (PMC2397393; doi:10.1186/1471-2148-8-142)
Supplement: Additional file 5 — Supplementary table 4. Accession numbers of intronic and pseudogenic sequences generated in this study. Code: Y1 for RPS4Y1, Y2 for RPS4Y2, Y for unique RPS4Y gene, and ψ for pseudogene. [file 1471-2148-8-142-S5.pdf]

Supplementary table 4: Accession numbers of intronic and pseudogenic sequences generated in this study. Code: Y1 for *RPS4Y1*, Y2 for *RPS4Y2*, Y for unique *RPS4Y* gene, and ψ for pseudogene.

Name	Species	Intron3 accession number	Intron6 accession number
Ptr Y1	*Pan troglodytes*	EF408708	EF408722
Ggo Y1	*Gorilla gorilla*	EF408709	EF408723
Ppy Y1	*Pongo pygmaeus*	EF408710	EF408724
Mfu Y1	*Macaca fuscata*	EF408711	EF408725
Msp Y1	*Mandrillus sphinx*	EF408712	-
Ggo Y2	*Gorilla gorilla*	EF408713	EF408726
Ppy Y2	*Pongo pygmaeus*	EF408714	EF408727
Mfu Y2	*Macaca fuscata*	EF408715	EF408728
Msp Y1	*Mandrillus sphinx*	EF408716	-
Sbo Y	*Saimiri boliviensis*	EF408717	EF408729
Cja Y	*Callithrix jacchus*	EF408718	EF408730
Cmo Y	*Callicebus moloch*	EF408719	-
Efu Y	*Eulemur fulvus*	EF408720	EF408731
Ema Y	*Eulemur macaco*	EF408721	-

Name	Species	Accession number
Ptr ψ	*Pan troglodytes*	EF408702
Ggo ψ	*Gorilla gorilla*	EF408703
Ppy ψ	*Pongo pygmaeus*	EF408704
Mfu ψ	*Macaca fuscata*	EF408705
Sbo ψ	*Saimiri boliviensis*	EF408706
Cja ψ	*Callithrix jacchus*	EF408707
