# Supplementary material for: RPS4Y gene family evolution in primates
Source: BMC Evol Biol. 2008 May 13;8:142. doi: 10.1186/1471-2148-8-142 (PMC2397393; doi:10.1186/1471-2148-8-142)
Supplement: Additional file 6 — Supplementary table 5. Accession numbers of nucleotide and protein sequences used from GenBank for cDNA and protein analyses. Y1 is used for RPS4Y1, Y2 is used for RPS4Y2, and X is used for RPS4X genes. [file 1471-2148-8-142-S6.pdf]

Supplementary table 5: Accession numbers of nucleotide and protein sequences used from GenBank for cDNA and protein analyses. Y1 is used for *RPS4Y1*, Y2 is used for *RPS4Y2*, and X is used for *RPS4X* genes.

Name	Species	cDNA Accession Number	Protein Accession Number
Hsa Y1	*Homo sapiens*	NM_001008.3	NP_000999.1
Ptr Y1	*Pan troglodytes*	AY633110.1	Q861U9
Ppa Y1	*Pan paniscus*	AH012490.1	Q861V0
Ggo Y1	*Gorilla gorilla*	AH012492.1	Q861U8
Ppy Y1	*Pongo pygmaeus*	AH012493.1	Q861U7
Mfu Y1	*Macaca fuscata*	D50105.1	BAA21076.1
Hsa Y2	*Homo sapiens*	NM_001039567.2	Q8TD47
Ptr Y2	*Pan troglodytes*	AY633111.1	Q6GVM7
Mfu Y2	*Macaca fuscata*	AB024286.1	BAA87933.1
Hsa X	*Homo sapiens*	NM_001007.3	AAH71662.1
Ptr X	*Pan troglodytes*	XM521131.1	-
Mfu X	*Macaca fuscata*	AB024285.1	Q76MY1
Cae X	*Chlorocebus aethiops*	AB015610.1	Q76N24
