# Supplementary material for: RPS4Y gene family evolution in primates
Source: BMC Evol Biol. 2008 May 13;8:142. doi: 10.1186/1471-2148-8-142 (PMC2397393; doi:10.1186/1471-2148-8-142)
Supplement: Additional file 1 — Additional figure 1: Primate RPS4 protein alignment. Four different Pfam domains are marked in different colors. Nucleotide positions under positive selection in the human RPS4Y2 lineage are highlighted in red squares. [file 1471-2148-8-142-S1.pdf]

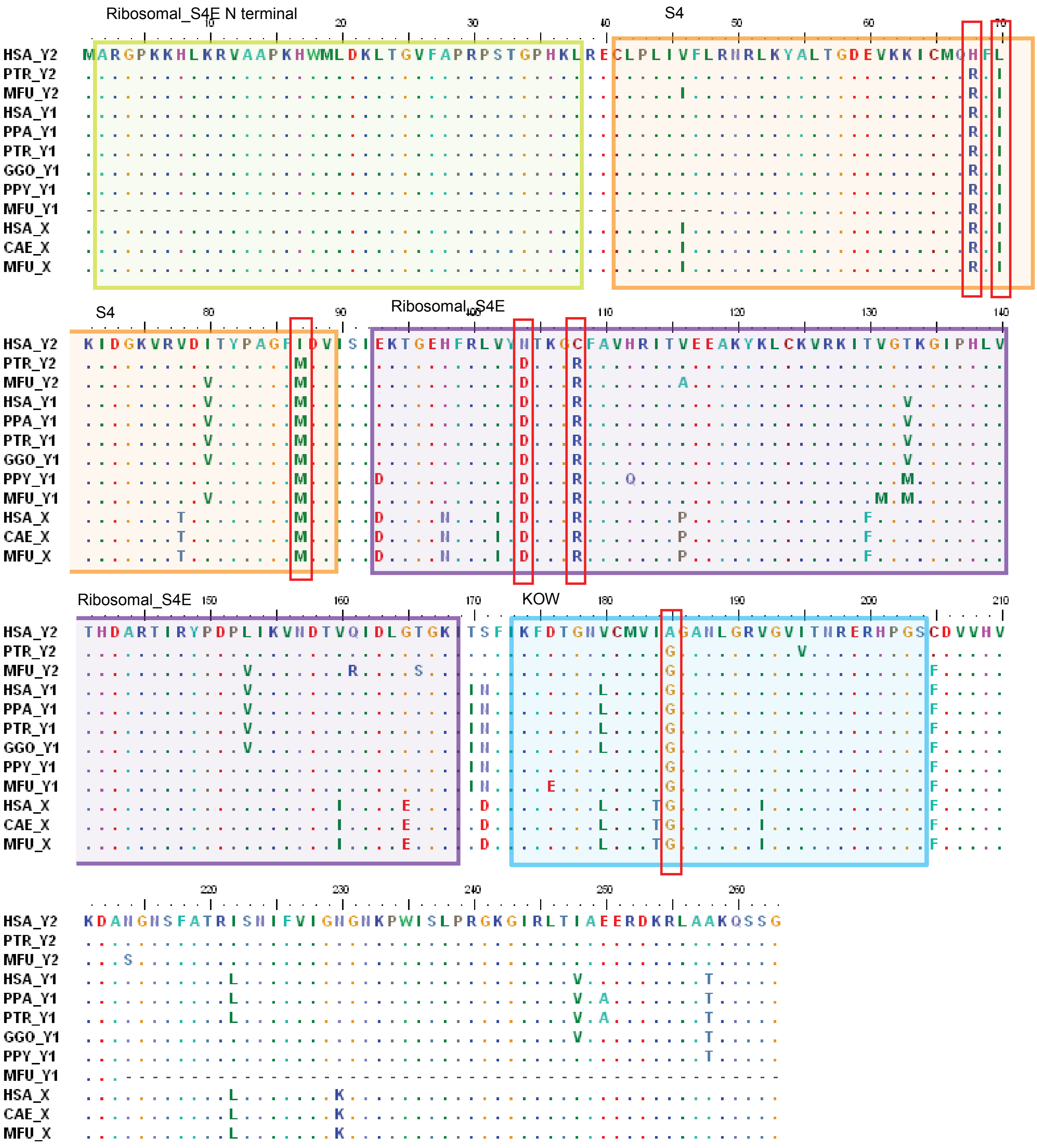

Ribosomal_S4E N terminal
S4
Ribosomal_S4E
KOW
HSA_Y2
PTR_Y2
MFU_Y2
HSA_Y1
PPA_Y1
PTR_Y1
GGO_Y1
PPY_Y1
MFU_Y1
HSA_X
CAE_X
MFU_X
